# The role of income in the lives of people with long-term disabilities: A multi-country analysis

**DOI:** 10.1093/eurpub/ckac131.110

**Published:** 2022-10-25

**Authors:** A Oña, D Pacheco Barzallo

**Affiliations:** 1 Health Services and Rehabilitation, Schweizer Paraplegiker-Forschung, Nottwil, Switzerland; 2 Department of Health Sciences and Medicine, University of Lucerne, Lucerne, Switzerland

## Abstract

**Background:**

Health is a fundamental human right. Yet, people become ill and die because they do not have enough money to access the necessary healthcare. Studies confirm that on average, more income translates to better health status. The magnitude of this relationship and its consequences beyond health like reintegration or spillovers are not widely understood but are expected to be significantly different among countries. This is especially the case in vulnerable populations like those with a spinal cord injury (SCI). This study aims to better understand the role that income plays in promoting health in people with SCI and their differences across countries.

**Methods:**

The research focus on three aspects, one, the estimation of the health income gap using years after the injury and a comorbidity index, two, the decomposition of different factors of this inequality with a special interest in the unmet healthcare needs, and three, their consequences in their social participation measured by the reasons of unemployment.

**Results:**

There are significant differences in health status by income and between countries. On average, the poorest income groups with SCI are up to 40 times more likely to be ill than people in the richest groups and live 4-6 years less after the injury. The working conditions and the unmet health care needs are important factors that explain that differences. In addition, the health status, the lack of sustainable employment opportunities, and workplace accessibility issues are the main issues to reintegrating into the labor market.

**Conclusions:**

Health inequalities are a global problem that affects people with long-term health conditions disproportionately to others without. Even in more equal and high-income countries with a universal health system, there are different realities for the people according to their income, where the estimations and solutions for the ‘average’ individual do not target the needs of people in the extremes.

**Key messages:**

• This study generates evidence about the role of income in health, lack of health services, and consequences in people with SCI across and within countries.

• To achieve the goal of health equity, it is important to target the different realities focusing on the needs of people who most need which goes beyond the health system and changes by income level.

